# *Sporothrix brasiliensis*: A Review of an Emerging South American Fungal Pathogen, Its Related Disease, Presentation and Spread in Argentina

**DOI:** 10.3390/jof7030170

**Published:** 2021-02-26

**Authors:** Alejandro Etchecopaz, María A. Toscanini, Amelia Gisbert, Javier Mas, Miguel Scarpa, Cristina A. Iovannitti, Karla Bendezú, Alejandro D. Nusblat, Ricardo Iachini, María L. Cuestas

**Affiliations:** 1 Cátedra de Enfermedades Infecciosas, Facultad de Ciencias Veterinarias, Universidad de Buenos Aires, Buenos Aires C1427 CWN, Argentina; aetchecopaz@fvet.uba.ar (A.E.); mvscarpa@gmail.com (M.S.); 2 Instituto de Nanobiotecnología (Nanobiotec), Consejo Nacional de Investigaciones Científicas y Técnicas (CONICET), Universidad de Buenos Aires, Buenos Aires C1113 AAD, Argentina; agus.toscanini@gmail.com (M.A.T.); anusblat@gmail.com (A.D.N.); 3 Cátedra de Clínica Médica, Facultad de Ciencias Veterinarias, Universidad de Buenos Aires, Buenos Aires C1427 CWN, Argentina; gisbertma@hotmail.com (A.G.); javiermas@diagnotest.com.ar (J.M.); 4 Centro de Micología, Instituto de Investigaciones en Microbiología y Parasitología Médica (IMPaM), Consejo Nacional de Investigaciones Científicas y Técnicas (CONICET), Universidad de Buenos Aires, Buenos Aires C1121ABG, Argentina; caiovannitti@yahoo.com.ar (C.A.I.); karlitabendezu@yahoo.com.ar (K.B.); 5 Instituto de Zoonosis «Luis Pasteur», Buenos Aires C1405 DCD, Argentina; r_iachini@yahoo.com.ar

**Keywords:** cat-transmitted sporotrichosis, zoonosis, *Sporothrix brasiliensis*, virulence factors, diagnosis, therapy, vaccines, Argentina

## Abstract

Sporotrichosis, caused by *Sporothrix schenckii* and related species, is the most frequent implantation mycosis in Latin America. In Argentina, over the last 8 years, there have been 0.16 new cases per month of feline sporotrichosis in 2011, increasing to 0.75 cases per month in 2019 and involving zoonotic transmission to humans. Molecular identification by polymerase chain reaction (PCR) detected *Sporothrix brasiliensis* in these feline and zoonotic outbreaks. This study will focus on different feline and human sporotrichosis outbreaks caused by *S. brasiliensis* in Argentina during 2011–2019. We will address the sources of infection and environmental hotspots, as well as the application of several treatment strategies for improving the pharmacotherapy of the different clinical forms of the disease. Finally, we will provide a detailed summary of the clinical aspects and new advances in host–pathogen interactions, virulence factors and immune response, focusing on state-of-the-art diagnostic tools and potential vaccine candidates.

## 1. Introduction

Sporotrichosis is a fungal implantation disease of subacute course caused by certain species of the *Sporothrix schenckii* complex, order Ophiostomatales, affecting the skin and lymph nodes. In recent years, sporotrichosis due to *Sporothrix brasiliensis* has emerged in Brazil as a zoonotic disease transmitted from infected cats to humans through bites, scratches or contact with exudate from the cutaneous lesions of sick cats [1]. This species appears to cause more severe disease among humans and animals than other *Sporothrix* species and is also less susceptible to itraconazole [2]. Only in the Brazilian state of Rio de Janeiro has this emerging disease reached hyperendemic proportions with more than 4000 human and feline sporotrichosis cases between 1998 and 2012 (>22.22 cases per month) [3].

The sporotrichosis epidemic is still active and a matter of concern in the Southeastern states of Brazil, where a progressive increase in the incidence and prevalence of this mycosis is observed, with more than 3510 human cases reported from January 2015 to May 2018 (68.82 cases per month) [4]. It is noteworthy that this zoonotic outbreak is spreading in Brazil and other neighboring countries, such as Argentina, where the incidence of this mycosis is on the rise with cat-transmitted sporotrichosis (CTS) being the most important route of infection, similar to the Brazilian scenario but to a much lesser degree [5,6].

The information on the prevalence rates of sporotrichosis due to *S. brasiliensis* in Argentina is scarce, almost reflecting an invisible burden. The aim of the present study is to give an overview of the different outbreaks of feline and human sporotrichosis in Argentina in the last 8 years and to contribute to the knowledge of this expanding epidemic.

We will summarize the latest research progress on the epidemiology, pathogenesis, and clinical characteristics of feline and human sporotrichosis caused by *S. brasiliensis* and discuss the current treatments and scientific advances to potentially combat this epidemic.

We will use the terms ‘proven, probable and possible’ to refer to CTS diagnosis throughout the text in accordance with the level of evidence for the diagnosis of zoonotic transmission of human sporotrichosis, recently proposed in the Guidelines for the Management of Human Sporotrichosis, Brazil, presented at the 18th Latin American Forum of Fungal Infections in Clinical Practice (INFOCUS = Meeting (2020) [7]. In brief, the entry criterion is a history of trauma or contact with sick cats. A proven diagnosis requires fungal isolation from lesions by culture. A probable CTS diagnosis is based on the presence of a combination of host factors, clinical features, and positive mycology (i.e., direct exam and/or histopathology with suggestive fungal elements). A diagnosis of possible CTS is made in the presence of host factors and clinical features but in the absence of mycological criteria. Cases that do not fall into one of these three categories are not considered CTS.

## 2. *S. Brasiliensis* and Sporotrichosis

Even though the first clinical case of cutaneous sporotrichosis was published in 1898 by Benjamin Schenck [8], the ascomycete *S. brasiliensis* was an unknown species until 2007 [9]. In other words, *S. schenckii* was the only known species implicated in this granulomatous disease until the early 2000s, when molecular studies allowed the description of additional species that include not only *S. brasiliensis*, but also *Sporothrix globosa*, *Sporothrix mexicana* and *Sporothrix luriei* [9]. Hence, *S. brasiliensis* is considered an emerging fungal pathogen that can be transmitted via a zoonotic (cat-to-human) and/or enzootic (cat-to-cat/cat-to-dog) mode with epidemic and/or epizootic potential [10].

*S. brasiliensis* is a geophilic thermally dimorphic fungus that exhibits a saprophytic mycelium phase at room temperature (25–28 °C) and a cigar-shaped yeast pathogenic phase at 36–37 °C [11]. It is a member of the pathogenic clade of the genus *Sporothrix,* together with *S. schenckii* sensu stricto, *S. globosa* and *S. luriei* and is prevalent in South America [12]. *S. brasiliensis* emerged in the late twentieth century and shows a preponderance for cat-transmission rather than for sapronotic transmission as in other *Sporothrix* species. Interestingly, a major difference between these two sources of transmission is the morphotype inoculated: hyphae and conidia in sapronoses and yeasts in CTS.

**Figure 1:**
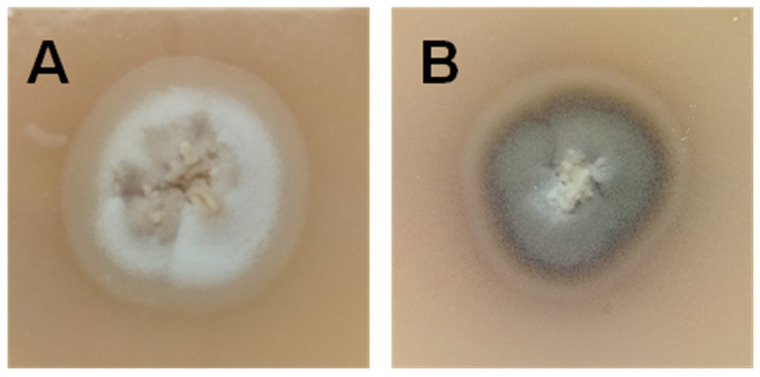
(**A**) Light” (or albino) and (**B**) “Dark” (or pigmented) *S. brasiliensis* phenotypes isolated from the face lesions and nail tips, respectively, of a sick cat from Argentina with the lymphocutaneous form of the disease.

According to the level of pigmentation or melanization of the environmental or mycelial form of *S. brasiliensis* in the mycological cultures, Sales et al. described two different phenotypes: the “Light” (or albino) and the “Dark” (or pigmented). Both phenotypes can differ in the in vitro susceptibility profiles to different antifungal drugs and might differ in the host immune response [13,14]. In addition, Oliveira et al. showed in a murine model of sporotrichosis that the “Light” phenotype caused more severe disease than the “Dark” counterpart [14]. Coinfection by both phenotypically different *S. brasiliensis* phenotypes have been described in domestic felines in the hyperendemic area of Brazil [13,14]. In Argentina, both phenotypes were also isolated from the face lesions and nail tips of a sick cat with the lymphocutaneous form of the disease (Figure 1).

It should be noted that these virulence-related phenotypes are variably expressed within other *Sporothrix* species [15,16].

In addition to these two phenotypes, we demonstrated the circulation of different *S. brasiliensis* strains with low, medium and high levels of virulence and also with the ability to disseminate across the central nervous system (CNS) [2].

The whole genome of *S. brasiliensis* comprises a size of 33.2 Mb with a G+C content of 62% and a number of 9091 protein coding genes (Genbank accession number AWTV00000000) [17]. Of them, 395 species-specific genes and 60 unique proteins were described [18].

**Figure 2:**
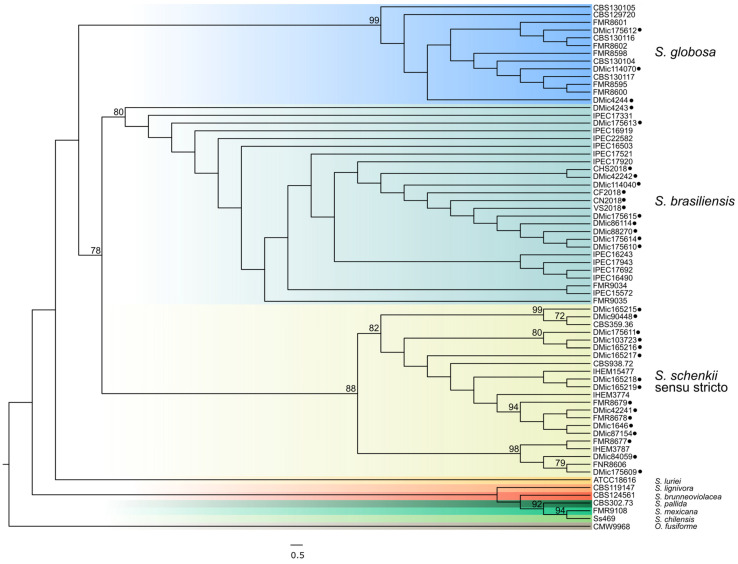
Phylogenetic tree generated from a maximum likelihood analysis based on the partial calmodulin sequence alignments of different *Sporothrix* spp. strains, including those from Argentina (black circles). The best model of evolution was estimated using the JModelTest software considering both the Bayesian and the Akaike information criteria and the phylogenetic tree was constructed using the PhyML 3.0 software. Clade stability was determined using a bootstrap resampling procedure with 1000 replicates. Gaps were treated as missing data. The nucleotide sequences of the corresponding genes of *Ophiostoma fusiforme* were used as outgroup.

Some genetic features related to the animal pathogenic life-style of this *Sporothrix* species (i.e., lack of plant cell wall degrading enzymes present in other soil-borne Sordariomycetes, and lack of expansion of peptidase genes) were noticed and suggest an evolutionary adaptation of *S. brasiliensis* from plant-association or saprobes to mammalian parasitism [17]. In this regard, phylogenetic data support a recent habitat shift within *S. brasiliensis* from plant to cat that seems to have occurred in southeastern Brazil that is responsible for its emergence [19]. It was also described that *S. brasiliensis* displays low degrees of genetic variability (i.e., low haplotype and nucleotide diversity with undetectable recombinant events), showing a high level of clonality among isolates throughout the Brazilian territory [19]. That is, *S. brasiliensis* has emerged as a clonal genotype during the zoonotic outbreak in Brazil. This clonality is also observed in both human and animal Argentinean isolates, which clustered together in a monophyletic clade (Figure 2).

Nowadays, sporotrichosis caused by *S. brasiliensis* is considered an important emerging mycosis with severe clinical forms in both immunocompetent and immunocompromised hosts. Immunological epiphenomena such as erythema nodosum, erythema multiforme, arthralgia, myalgia, arthritis, retinitis were also described in CTS by *S. brasiliensis* [20]. Since the first recorded observations around the year 2000 of cat-transmitted *S. brasiliensis* infections in Rio de Janeiro, Brazil, more than 12,000 cases had been recorded in that state up to 2017. As of 2018, CTS cases have been documented in at least 10 Brazilian states, including Sao Paulo, Rio Grande do Soul, Rio Grande do Norte, Mato Grosso, Minas Gerais, Paraná [21]. Some of these states, such as Rio Grande do Soul and Paraná share borders with Argentina, where this zoonosis is expanding. 

It was not until 2015 that the first *S. brasiliensis* proven human cases outside Brazil were reported. There were four cases of patients living in the Northwest of the city of Buenos Aires that had contact with infected domestic cats between 2011 and 2014 [22]. However, a retrospective study conducted by Cordoba et al. in 2018 demonstrated that the first *S. brasiliensis* proven CTS case occurred in 1988, from a patient scratched by a cat [23]. In 1990, a case of an 8-year-old girl who presented with an ulcerated lesion of the right mandibular region, following a cat scratch was also reported. *S. schenckii* was isolated in culture from the ulcerated lesion, although it is uncertain if this isolate was *S. brasiliensis* as a molecular analysis was not performed [24]. It should be noted that this case had been previously misdiagnosed as tuberculosis and leishmaniasis [25]. Hence, *S. brasiliensis* is not geographically restricted to Brazil, as previously described [25], and it is spreading to other South American countries. In Paraguay in 2017, two cases of probable *S. brasiliensis* were reported in sick cats brought from Brazil by their owners [26]. Suspected or possible cases were detected in Bolivia, Colombia and Panama [1,27]. It should be remarked that in the Brazilian scenario of feline sporotrichosis, *S. brasiliensis* is the most prevalent agent and primary pathogen among cats (96.8%), while *S. schenckii* was identified only once [10]. However, cases of feline sporotrichosis due to *S. schenckii sensu stricto* were recently described in Malaysia [28].

There are still many missing pieces in the puzzle to completely understand the unique epidemic scenario caused by *S. brasiliensis*, such as the high susceptibility of cats to this fungal species, the high fungal virulence possibly related to its recent introduction into urban feline populations, and how feline sporotrichosis has emerged. Cats´ lifestyle is not a minor issue and their habits might partly explain their role as sporotrichosis transmitters: roaming, peridomicile, nail sharpening in the environment, toileting in contact with soil, mating behavior, and territorial disputes that usually lead to bites and/or scratches spreading the fungus to other susceptible hosts [29]. In addition, cats are among the most common pet animals with close contact to humans and the major predators of rats. In this regard, Lutz and Splendore demonstrated that rats can acquire sporotrichosis by ingestion [30]. Hence, a hypothesis is that cats were infected with *S. brasiliensis* by rat ingestion and that the fungus was able to adapt to cat saliva. It should be mentioned that animal saliva has an “environmental” condition similar to that of fermented plant material, reinforcing the theory related to the host shift of *Sporothrix* from plant to animal (feline body temperature of 37.7–39.1 °C, and cat saliva pH of 7.5–8.0). Even though *Sporothrix* species are not plant pathogens, they require for promoting their growth some particular conditions present in the decaying plant material, which are similar to those obtained during advanced decomposition and fermentation, such as high temperature and humidity. These conditions might lead to metabolic changes and oxidative stress that might induce a morphological shift that favors the invasive yeast growth [31].

On the other hand, the steady rise in temperature upon climate change selectively enables the adaptation of fungi, broadening the array of species able to survive at host temperature. This feature is necessary for virulence and pathogenesis, since a major dominant trait of all human and mammal pathogenic fungi is the ability to grow at host temperature. Hence, global warming and the accompanying climate changes may be partly responsible for the drastic increased incidence of sporotrichosis and many other emerging fungal diseases.

## 3. Sporotrichosis Outbreaks in Argentina and the Hidden Burden: Lessons to Learn About Detecting, Controlling and Preventing Exposure

In Argentina, sporotrichosis is, along with chromoblastomycosis, the second most common subcutaneous mycosis after mycetoma [32]. A recent epidemiological and molecular study carried out by Cordoba et al., described the circulation of *S. schenkii sensu stricto*, *S. brasiliensis* and *S. globosa* in Argentina, since at least 1984 [23].

Despite the clinical importance and the confirmed circulation of these three pathogenic species in the region, there are no data in Argentina regarding either the incidence or prevalence of sporotrichosis. Only few clinical cases have been reported [5,6,22,24,33,34,35,36,37,38].

However, no data does not mean non-existence and sporotrichosis might be more widespread than previously thought. The real burden of the disease is still unknown due to the lack of epidemiological studies, notification and awareness of this fungal disease by many healthcare professionals and pet owners, noting the importance for veterinarians and clinicians to begin to recognize—and even become acquainted with—this implantation mycosis and zoonotic disease. The pathogen *S. brasiliensis* is there and should be included in the diagnostic algorithms of many human and cat diseases (such as leishmaniasis and tuberculosis in humans or squamous cell tumors in cats), as discussed below.

**Figure 3:**
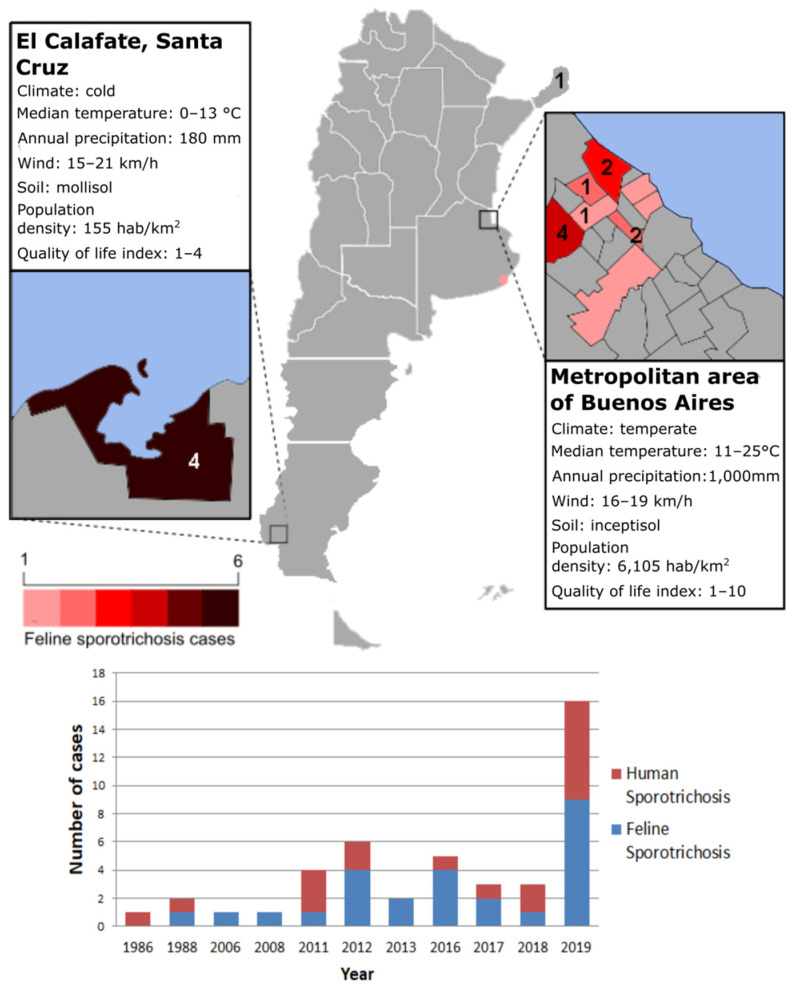
Distribution patterns and frequencies of *S. brasiliensis* in Argentina.

The earliest detection of *S. brasiliensis* in Argentina came from a retrospective sampling of human and animal clinical isolates from Misiones province (a province bordering Brazil) in 1986 and then in Buenos Aires province in 1988 [23]. Since then, 21 cases of human sporotrichosis and 24 of feline sporotrichosis have been diagnosed by our research group or reported in the literature, reaching unprecedented levels, with a four-fold increase of *S. brasiliensis* infections in humans from 2011 to 2019. Most of them occurred in the Northern area of Buenos Aires province; however, in El Calafate, Santa Cruz province, an outbreak of CTS occurred in 2019 [39]. According to Ramírez-Soto et al., medically important *Sporothrix* spp. grows in soil at temperatures of 6.6. °C to 28.84 °C and 37.5% to 99.06% relative humidity [40]. Therefore, the southern town of El Calafate has the environmental conditions for *S. brasiliensis* growth. In Buenos Aires, most cases occurred in the Northern area of the province bordering the Reconquista River basin (Cuenca del Rio Reconquista) (Figure 3), indicating that this region is the hotspot of this emerging fungal disease.

Among the humans with sporotrichosis related to *S. brasiliensis* or to CTS, 76.2% of them (*n* = 16) had a positive culture and reported scratches, bites or contact with exudates from sick cats with sporotrichosis. In all cases, except two, a molecular diagnosis could be performed demonstrating the presence of *S. brasiliensis*. One patient (4.7%) was infected by traumatic inoculation of a pine wood splinter, and another one (4.7%) reported a rat bite. The most common clinical forms of sporotrichosis in this studied population were the lymphocutaneous (*n* = 9) followed by the fixed cutaneous forms (*n* = 4), as described in the literature. The median age was 34.5 years (range 3–67) and 58.8% were women with a mean age of 32.5 years old. The main clinical features of the human cases with sporotrichosis due to *S. brasiliensis* are depicted in Table 1. Due to the retrospective nature of this study, not all data are available for every human case.

With regard to the cases of feline sporotrichosis (Table 2), most of them were domestic cats with a median age of 2.6 years (1.5–10) with free access to the street. Most of them were males (60.8%) and were identified as of undefined breed (95.6%). Only two cats were Siamese (8.7%). During the study, five cats died from a fatal systemic form, six cats received treatment with itraconazole (10–50 mg/kg/day) for at least 2–3 months and were clinically cured. No data are available of the clinical outcome of the remaining 13 cats. In seven cases, the molecular characterization of *Sporothrix* could be performed, confirming the involvement of *S. brasiliensis* in these feline cases of sporotrichosis. In another 10 cats, the epidemiological nexus were human cases with sporotrichosis caused by *S. brasiliensis* who were bitten or scratched by these sick animals.

**Figure 4:**
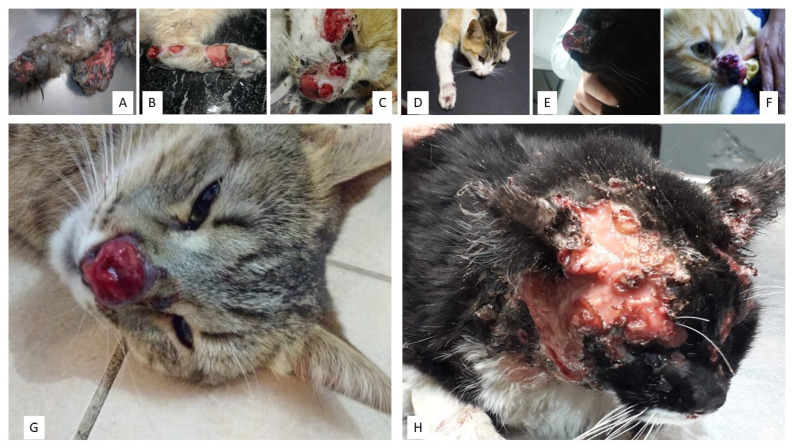
Feline sporotrichosis. (**A**) Multiple ulcerative crusted lesions around the claws. (**B**) Multiple ulcerative crusted lesions on the hind limbs. (**C**) Ulcerative crusted facial lesions. (**D**) Ulcerative lesion on the forelimbs. (**E**–**G**) (Multiple) ulcerative crusted lesion on the nose. (**H**) Multiple ulcerative crusted lesions on the head.

Cutaneous lesions in cats were comparable with those previously described in different cat populations from Brazil [41], including crust, ulceration and presence of pus and blood, as well as respiratory signs and weight loss, with sneezing being the most frequent. Most cutaneous lesions were localized on the head, especially the nose and ears, and in a few cases on limbs and tail (Figure 4). 

Currently it is very difficult to determine the scale of feline epizootic sporotrichosis because the report of this disease is not officially required. Since 2020, the notification has only been mandatory in Buenos Aires province, but not in other provinces. Hence, the real prevalence of feline sporotrichosis, and certainly its real epidemiological importance, might be underestimated. There are also no data available from stray feline cases. In addition, the numbers presented herein represent mostly those of feline cases registered at the School of Veterinary Medicine (University of Buenos Aires, UBA, Buenos Aires, Argentina), the Center of Mycology of the School of Medicine (UBA), the Department of Mycology at the “Dr Carlos G. Malbrán” Institute, Buenos Aires and the Institute of Zoonoses, Buenos Aires. Strikingly, misdiagnosis is frequent because it is not included in the differential diagnosis of many cutaneous diseases. Moreover, cytology/histopathology are not common practices among local veterinarians and the social and economic condition of many pet owners living in social-environmental vulnerability prevents them from affording veterinary consultations or even attending their offices. Furthermore, veterinarians do not frequently wear personal protective equipment, such as gloves, when handling cats. This should be changed. Veterinarians and cat owners should always wear gloves when handling, examining, healing or collecting clinical samples of exudates and tissues of suspected animals and should avoid bites and scratches. The panorama is even bleaker when most cases of ulcerative lesions in cats are clinically misdiagnosed as squamous cell tumors, which is much more common than might be thought, the solution in many cases being euthanasia. Hence, cats are often euthanized because this fungal disease is not included in the diagnostic algorithm used by a significant proportion of veterinarians when dealing with a sick cat exhibiting cutaneous “sporotrichoid” lesions in the nose, ears and eyelids. If accurate sporotrichosis diagnoses were performed, many cat lives could be saved. 

Hence, veterinarians and clinicians should be trained and must consider a possible *Sporothrix* sp. infection in endemic countries when dealing with complex problems such as cancer, acquired immunodeficiency syndrome (AIDS), critical care and respiratory diseases, or when cutaneous lesions are similar to those described for sporotrichosis. Diagnoses should be accurately made and an empiric treatment should be the exception and not the rule. Laboratory training is also critical to make an accurate diagnosis [42]. Veterinarians should also advise cat owners of a sick pet with sporotrichosis about the high risk of zoonotic transmission.

At present, Argentinean environmental isolates of *S. brasiliensis* were recovered only from soil in two nine-banded armadillo caves in Chaco province in 2003 [23]. Soils associated with pine might be another source of *S. brasiliensis*, since many of the cats with sporotrichosis by *S. brasiliensis* were roaming cats rescued from abandoned old houses with dirty pine wood floors. It is noteworthy that a human case of lymphocutaneous sporotrichosis by *S. brasiliensis* in an immunocompetent 22-year-old man, a woodworker in the locality of Moreno, Buenos Aires province, who acquired the fungus via the traumatic inoculation of a pine wood splinter on the dorsum of his right wrist, has recently been documented. He described an inoculating event before the onset of the lesion with a pine wood splinter, reporting no previous contact with cats. 

Thus, a potential association between pine wood and sporotrichosis due to *S. brasiliensis*, should be considered. Particularly, this type of material contains a wide diversity of arthropods, whose abundance and biomass are high in the bark structure [43]. Among them, arthropods belonging to the order Cleoptera such as beetles have been described assessing the bridge linking the ecology of human pathogens in *Sporothrix* to that of beetle-related ascomycetes of the genus *Ophiostoma* as was previously described [12]. In addition, it has been demonstrated that *Sporothrix* virulence factors might have evolved from interactions during environmental growth [44,45]. In this regard, we should mention the old outbreak of sporotrichosis in Witwatersand, South Africa, in the 1940s, where infection was associated with *Sporothrix* on wooden props used in gold mines [46]. All these findings reinforce the idea that human infections with *S. brasiliensis* do not occur exclusively via zoonotic transmission as has been previously reported [1].

On the other hand, sporotrichosis has long been recognized as an occupational disease, formerly recognized as the gardener´s disease or rose disease. However, in the case of *S. brasiliensis*, veterinarians, veterinary technicians, animal caretakers, cat owners and woodworkers or professionals handling wood have become the novel risk groups of sporotrichosis due to this emerging fungal species.

Unfortunately, sporotrichosis has a future in at least Argentina and Brazil. Although there is a need for epidemiological research to measure the real burden of disease in various regions of South America, actions can, and should be taken to diagnose and treat patients and cats accurately. There is a founded need to develop standard mycological practices in the area to promote an early clinical diagnosis, as well as to develop new rapid diagnostic tools and advocate for an affordable treatment. New, simple, reliable and affordable diagnostic assays are presently being tested in Brazil. These novel diagnostic tools will improve the knowledge of local epidemiologies and reduce cat transmission to humans and other mammals through early diagnosis and increased awareness. 

Finally, as stated by the Global Action Fund for Fungal Infections (GAFFI) “To gain a full picture of the incidence, prevalence and trends over time of fungal diseases—in this case, sporotrichosis — local, regional and national studies are critical” [42].

## 4. Sporotrichosis: An Indicator of Socio-Environmental Vulnerability?

Despite the influence of socio-environmental factors in infectious diseases having been widely documented, this phenomenon remains unclear regarding mycoses. Alzuguir et al. reported that social and geoecological factors can affect the incidence and prevalence of fungal diseases [47]. Global warming and changes in humidity and rainfall can be potent cofactors leading to changes in the fungal phenotypes allowing their adaptation, and the emergence and spread of disease, as occurs with viral diseases such as dengue, zika and chikungunya, explaining in some way the Patagonian outbreaks of sporotrichosis. Moreover, human activity can also contribute to the emergence of fungal diseases by modifying natural environments. 

It should be noted that the feline sporotrichosis epidemic started in Rio de Janeiro State, Brazil, in areas of low socioeconomic status and, precarious sanitary and health system, with high demographic density of both human and feline populations, as happens in the localities of the province of Buenos Aires where most of the outbreaks occurred [48,49].

Sporotrichosis has thus become an urban problem with a peridomicile character also in Argentina. Social vulnerability of the patients affected and lack of adequate information for the population are key factors in the comprehension of the spread of this epidemic. Better-trained personnel for the diagnosis of this fungal disease and access to the adequate antifungal therapy are mandatory in countries where the disease is endemic. 

It should be pointed out that data from Brazil, and from Argentinean areas with sporotrichosis outbreaks due to *S. brasiliensis*, showed a negative correlation between this emerging disease and socio-economic data regarding population, demographic density, human development index, health facilities and sanitary sewage. Hence, data suggest that this emerging disease represents an important indicator of socio-environmental vulnerability in Argentina and Brazil. The adoption of measures to mitigate the social and environmental impact on this fungal disease, especially in those municipalities with low socio-economic status, is crucial. Despite the high lethality in untreated cats and untreated immunosuppressed patients, sporotrichosis, just as with other better-known fungal diseases, is not a national notifiable condition in many geographical endemic regions for this disease. Fortunately, in May 2020, in Buenos Aires province, sporotrichosis due to *S. brasiliensis* was included as a notifiable disease at least in small animals.

## 5. *S. Brasiliensis* Interactions with the Human Host: Fungal Virulence Factors and Host Immune Response

### 5.1. Virulence Factors

One of the major differences between *S. brasiliensis* and *S. schenckii* is the high virulence observed in the former [49,50]. Some virulence factors are shared among the different *Sporothrix* species. However, others can be greatly related to the high virulence of *S. brasiliensis*, as will be described in this section.

The fungal thermal dimorphism exhibited by *S. brasiliensis* is essential for the establishment of infection and a result of convergent evolution [17]. This complex process is highly regulated by several genes, whose putative orthologs previously described in other dimorphic fungi including *Histoplasma capsulatum* (*ryp1, ryp2, ryp3, vea1*) and *Talaromyces marneffei* (*pakB, hgrA*) were also found in the recently sequenced genome of *S. brasiliensis*. Rodrigues et al. reported that the efficient transmission of *Sporothrix* yeast cells might be related to the more severe forms of the disease, hence hypothesizing that the yeast form is more virulent than its mycelial counterpart [51]. To further reinforce this notion, it was demonstrated that human peripheral mononuclear cells differentially recognized the different morphotypes of *S. brasiliensis* (conidia, yeast-like cells and germlings) due to changes in their cell wall composition. This differential recognition might influence the contribution of immune receptors during cytokine stimulation by human monocytes [52].

It has been recently demonstrated that *S. brasiliensis* in filamentous form can develop biofilms in vitro. Mature biofilms have been described as a dense extracellular polymeric matrix with conidia and hyphae embedded in their structure, with channels of hydration and distribution of nutrients. Biofilms decrease the effectiveness of antifungal drugs [53].

The cell wall is an essential structure for cell viability, interaction with the environment and the host immune cells and contains several macromolecules and pathogen-associated molecular patterns (PAMPs) involved in virulence, host recognition and immune response.

The cell wall of *S. schencki* is mainly composed of β-glucans, chitin and peptide-rhamnomannan (PRM). β-1-3 and 1-6 glucans are both found in pathogenic and non-pathogenic species and are widely recognized PAMPs among fungi, involved in the innate immune response. In contrast to *S. schenckii*, *S. brasiliensis* exhibits in its yeast parasitic form a thicker bilayer cell wall with greater rhamnose and chitin contents and longer cell wall microfibrils that can attach yeast cells to form biofilms [54]. These structural differences may affect their uptake by monocyte-derived human macrophages [54]. Distinct from those of other dimorphic fungi, the cell wall of *Sporothrix* spp. lacks α-glucan component. However, glycogen α-particles were identified in the cytoplasm of these fungi, close to the cell wall and the plasma membrane [54].

One of the major components of the *Sporothrix* spp. cell wall is the PRM, a molecule that harbors the O-glycan chains that are exclusive of *Sporothrix* spp. The PRM contains the main epitopes recognized by specific human serum IgG antibodies of patients with sporotrichosis [55]. Among them, a 60–70 kDa glycoprotein (3-carboxymuconate cyclase), which is important in the adhesion to the extracellular matrix [56,57] and a 47-kDa enolase (2-phospho-d-glycerate hydrolase), which is a metalloenzyme predicted to be an adhesin by the Fungal RV database [58,59]. Both of them are considered potential vaccine candidates for feline sporotrichosis prevention [58]. The former immunodominant antigen is associated with more virulent isolates of *Sporothrix*, particularly *S. brasiliensis* [58] and may have a key role in immunomodulation and host response. In this regard, Castro et al. reported that the highly virulent *S. brasiliensis* isolates showed reduced levels of cell wall gp60-70, observations that were confirmed by the topographical location of the gp60-70 antigen using immunoelectromicroscopy [49]. Cell wall lipids are also able to inhibit phagocytosis [60], and yeasts of *Sporothrix* are more virulent than longer-term culture conidia, possibly reflecting an enhanced evasion of phagocytosis [61].

Extracellular vesicles (EVs), including exosomes, microvesicles and apoptotic bodies, are lipid bilayer structures released by cells which carry different components that can affect many biological processes. EVs were reported in a myriad of organisms, including fungi (i.e., *H. capsulatum, C. albicans, P. brasiliensis*, *Cryptococcus neoformans* [62]. In fungi, these lipidic structures are associated with the transport of molecules across the cell wall along with the delivery of RNA, lipids, polysaccharides, proteins and immunoreactive components [62,63]. All these components can interact with host immune cells, contributing to drug resistance and facilitating cell invasion and pathogenesis [62] as is the case of *C. neoformans*, whose EVs are capable of crossing the blood–brain barrier increasing fungal damage in the brain [64].

Recently, EVs were reported in *S. brasiliensis* as important virulence factors that induce an increase in fungal burden *in vivo* after their inoculation into BALB/c mice before subcutaneous infection with yeast [65]. Results also showed a potential modulation of the host immune system in EVs-inoculated mice by modulating dendritic cells in vitro (increase in phagocytosis but without displaying good antifungal activity, and increase in interleukin-12 (IL-12) and tumor necrosis factor alpha (TNF-α), leading to opposite effects in the regulation of the immune response). By proteomic analysis, Ikeda et al. described that EVs from *S. brasiliensis* carry important virulence factors and antigenic components that can interact with the host immune system leading to the establishment of this fungal disease [15].

Other phenotypic characteristics associated with virulence are melanization, thermotolerance (which is fundamental for survival and parasitism) and the production of proteases, catalase and urease [15]. It was shown that the expression of melanin, urease and proteases in *S. brasiliensis* is higher than in *S. schenckii*. However, these two species display similar thermotolerances [15]. Melanin is a pigment present in the fungal cell wall that protects the fungus against harsh environmental and parasitic conditions by providing resistance to oxidizing agents. Both morphologic forms of *Sporothrix* spp. are capable of producing the three different types of melanin (DHN-melanin, eumelanin and pyomelanin). The former is the main pigment produced by these fungal species and is synthesized via acetyl-coA originated from glycolysis and the latter two via specific precursors, such as 3,4-dihydroxy-L-phenylalanine (L-DOPA) or L-tyrosine, respectively [66,67,68]. It has been reported that *Sporothrix* melanins protect the fungus against amphotericin B, terbinafine and nitrogen-derived oxidants [69]. The production of melanin is also associated with resistance to phagocytosis in the more pathogenic isolates of *Sporothrix*, notably to *S. brasiliensis* [15].

Other proteins with a potential impact on fungal virulence in *S. brasiliensis* were recently described by Rossato el al. and include: extracellular cell wall glucanase, aminopeptidase I, Mn superoxide dismutase, heat shock 70-kDa protein 1/8, glyceraldehyde-3-phosphate dehydrogenase (GAPDH), hydroxymethylglutaryl-coenzyme A (HMG-CoA) lyase, progesterone binding protein, rhamnolipid biosynthesis 3-oxacyl-(acyl-carrier-protein) reductase, and acetyl-CoA hydrolase [18]. Table 3 summarizes the different virulence factors described for *S. brasiliensis*, their function and cell localization.

### 5.2. Host Immune Response 

The immune response of susceptible hosts to control infection and disease caused by species has been scarcely studied. All these fungal species display distinct potential antigenic molecular components.

Innate immunity plays a role in the development of the adaptive response, by recognizing fungal wall elements through patron recognition receptors such as Toll-like receptors and C-type lectin receptors. These receptors are crucial in the activation of phagocytic effector functions and the induction of adaptive T helper (Th) responses, especially Th1 and Th17 [60]. Among Toll-like receptors (TLRs), TLR-2 and TLR-4 were shown to be important for the recognition of *S. brasiliensis* by macrophages, the production of optimal levels of interferon (IFN)-Ɣ, TNF-α, IL-6, IL-10, and nitric oxide. In contrast, their absence was shown to promote the persistence of infection in murine models of disseminated sporotrichosis [70,71]. Furthermore, Mario et al. reported that *S. brasiliensis* is more resistant to the peroxide stress associated to the antimicrobial mechanisms of phagocytes (i.e., reactive oxygen species, ROS) than *S. schenckii*. In addition, in vivo studies showed that *S. brasiliensis* was able to produce the highest levels of oxidative stress among the species of the *S. schenckii* complex (low catalase enzyme activity, low levels of thiols and glutathione), thus contributing to its highest pathogenicity and tissue damage [50]. Dendritic cells (DCs), macrophages and neutrophils are central in the mechanisms of fungal elimination either by phagocytosis or ROS production. All these immune cells are key during the establishment of a protective anti-*Sporothrix* response. In contrast, the adaptive branch of the immune response, particularly CD4+T cells, plays a major role in controlling dissemination of *Sporothrix* infection. Differentiation of CD4+T cells along a T-helper (Th) cell type 1 (Th1) or type 2 (Th2) pathway and development of specific Th responses determine the host´s susceptibility or resistance to invasive or disseminated fungal infections. The host protective immunity to fungal infections seems to rely largely on a Th1-biased response and this Th1 response may be beneficial against sporotrichosis [72]. Batista-Duharte et al. recently reported that in murine models of sporotrichosis, *S. brasiliensis* induced a more severe, disseminated and long-lasting disease associated with poor stimulation of the innate immune system in the skin and subcutaneous tissue and a reduction of the Th1 response with higher TH17 and Treg profile in the advanced phase of the infection that reduced the inflammatory response [73].

Cell-mediated immunity is also thought to play an important role in the control of feline sporotrichosis, since increased percentages of CD4 cells correlate with single lesions, well-organized inflammation, and lower fungal burden. In contrast, cats with the most severe forms of sporotrichosis display lesions with poorly formed granulomas and high fungal burden, which generally correlate with CD8low cell subsets triggered by Th2-cytokines [20,74].

## 6. Getting Sporothrichosis on the Map of Different Hosts

*Sporothrix* propagules usually enter the warm-blooded host through minor cutaneous trauma from contaminated plant debris or through scratches or bites from animals (mostly felines) carrying the fungus. Direct contact with exudates from cutaneous lesions of sick cats and droplet exposure in mucous membranes were also described [1]. Even though sporotrichosis is widely known as an implantation disease caused by traumatic inoculation, there have been some cases reported of inhalational sporotrichosis, particularly in the immunocompromised host, leading to disseminated pulmonary disease [75]. However, zoonotic transmission is considered the main mode of transmission of this emerging fungus to humans [1]. Although the most common types of sporotrichosis are the lymphocutaneous and fixed cutaneous forms which are restricted to the site of infection, atypical, extracutaneous and disseminated forms have also been reported, mostly in immunocompromised individuals as well as in felines, which will be described below. Noteworthy, these severe extracutaneous forms of the disease are more common with *S. brasiliensis* than other *Sporothrix* species.

### 6.1. Sporotrichosis in Immunocompetent Patients

Sporotrichosis in the immunocompetent host can present with a wide range of clinical manifestations from the fixed cutaneous form where the fungus remains localized to the implantation site, which is frequently the lower and upper limbs, to the extracutaneous forms with fungal dissemination to several organs and systems, such as bone and CNS [11] (Table 4). These severe forms are generally associated with host immunity and inoculum size, but also thermotolerance and virulence of the *Sporothrix* species. In this regard, among all *Sporothrix* species, *S. brasiliensis* is associated with more severe cases of the disease and/or atypical manifestations in both the immunocompetent and immunocompromised host [76]. For instance, immunoreactive (hypersensitivity reactions), meningeal, ocular, disseminated forms and sepsis of cutaneous focus are frequently associated to *S. brasiliensis* infections rather than to other *Sporothrix* species.

Nearly all reported human infections with *S. brasiliensis* are caused by the bites, scratches or contact with exudates from the cutaneous lesions of sick cats.

Feline lesions may harbor a high yeast burden, making *S. brasiliensis* unique for its ability to be transmitted in the yeast form, in contraposition to other *Sporothrix* species and even to other thermodimorphic fungi whose infective phase is the mycelial form. 

Ocular sporotrichosis is rarely reported and typically limited to the eyelids and eyebrows with regional lymphadenopathy following traumatic inoculation or as fixed-cutaneous ulceration or conjunctivitis following exposure [77,78]. Intraocular (endophthalmitis, retinal granuloma, granulomatous necrotizing retinochoroiditis, granulomatous uveitis) or extraocular (tissue adjacent to the bulb and ocular adnexal involvement) sporotrichosis were described after direct ocular traumatic inoculation or after hematogenous dissemination from other lesions [77]. Involvement of the nasolacrimal duct and lacrimal gland might also occur. 

Interestingly, in the last few years, the number of ocular sporotrichosis cases has increased, particularly in Rio de Janeiro, Brazil, where sporotrichosis has reached hyperendemic proportions [78,79,80,81,82,83]. The fungus can be isolated from the conjunctival secretion. In most cases a delay in the diagnosis was reported, leading to a greater chance of disease transmission and greater sequels for the affected patients. Indeed, after successful antifungal therapy, poor final visual acuity due to the scars left by the suppurative process can be an undesired effect of the disease [80].

Hypersensitivity reactions were reported as a manifestation of CTS in 4.5% of cases, but not as a manifestation of sporotrichosis by non-*brasiliensis* species [76]. This cell-mediated immune response was described to be similar to dermatophytid reactions, which are inflammatory reactions to a fungal infection at distant body sites [76].

Cases of chronic meningitis and hydrocephalus due to *S. brasiliensis* in immunocompetent adults were reported and mimic chronic meningitis due to *Mycobacterium tuberculosis*, thus an accurate diagnosis becomes a real challenge [84].

According to the Infectious Diseases Society of America, the first line therapy for fixed cutaneous, lymphocutaneous and osteoarticular cases of sporotrichosis is itraconazole (200–400 mg/day orally) [85]. Terbinafine in a dose of 500 mg twice daily may be used for fixed cutaneous and lymphocutaneous forms if itraconazole is contraindicated. If both antifungal drugs are not available, a saturated solution of potassium iodide can be used, although is not well tolerated and is associated with thyroid dysfunction. In the case of severe pulmonary symptoms, CNS, ocular or disseminated sporotrichosis, amphotericin B 50 mg/day is recommended. According to the reports, posaconazole might be an effective drug in combination with other antifungals for the treatment of severe disseminated forms of the disease [86,87].

### 6.2. Sporotrichosis in Immunocompromised Patients

Even though the most severe forms of sporotrichosis (cutaneous-disseminated, and extracutaneous forms with disseminated sporotrichosis, pulmonary sporotrichosis, and several osteoarticular, ocular and CNS disorders) are particularly associated with human immunodeficiency virus (HIV)/AIDS, other underlying diseases or comorbidities associated with immunosuppression, such as diabetes mellitus, malnutrition, drug abuse, chronic alcoholism, steroids, anti-TNF therapy, hematologic cancer and transplanted patients, should be considered [75,88].

CNS sporotrichosis presented as chronic meningitis or as part of the widespread dissemination has recently been increasingly reported in HIV/AIDS patients as part of the cat-associated epidemic of sporotrichosis in Rio de Janeiro State, Brazil. Mortality due to hydrocephalous complications were also reported [87].

In this regard, Freitas et al. reported that in the case of patients with both HIV and sporotrichosis in Brazil, approximately 58.3% of them exhibited the disseminated or disseminated cutaneous forms, rather than the localized, lymphocutaneous or fixed forms (41.7%) [89]. Low CD4 cell counts (≤200 cells/mm^3^) are strongly associated with disseminated and cutaneous-disseminated forms of the disease [90]. Since some of these patients with sporotrichosis and HIV coinfection might develop meningitis or meningoencephalitis with CNS invasion, lumbar puncture should be a practice in this group of immunocompromised patients to exclude this clinical entity in those immunosuppressed patients living in endemic or hyperendemic sporotrichosis areas. Freitas et al. observed that *S. brasiliensis* infection behaves similarly to *C. neoformans* infection, being neurotropic in humans, although the mechanisms implicated in CNS invasion and persistence are not yet completely understood [89]. Early diagnosis and successful therapy are challenging. The immune reconstitution inflammatory syndrome (IRIS) was associated with a more complicated course of sporotrichosis among HIV-infected patients, with a paradoxical clinical worsening after starting antifungal therapy [91].

Another severe and atypical presentations of CTS due to *S. brasiliensis* in immunocompromised patients included sporotrichosis of the mucosal surfaces of the mouth and nose that might lead to the destruction of the nasal septum, soft palate and uvula [90] and a meningeal and multiorgan disseminated sporotrichosis [92].

These findings highlight the potential aggressiveness of *S. brasiliensis* in immunocompromised patients, particularly in those with HIV/AIDS, living in endemic areas for sporotrichosis and the necessity to include this fungal disease in the differential diagnosis of all mentioned severe clinical forms associated with poor prognosis. 

### 6.3. Sporotrichosis in Pregnant Women

At present, 21 cases of sporotrichosis during pregnancy were reported in the literature during the sporotrichosis epidemic in Rio de Janeiro, Brazil, and the majority of cases were associated with CTS [93,94,95]. Of them, 20 showed the lymphocutaneous clinical presentation and the remaining one the cutaneous-fixed form.

Therapy during pregnancy is challenging due to the potential teratogenicity of many antifungal drugs. During the third trimester of gestation with few cutaneous lesions, thermotherapy is an acceptable alternative. During postpartum and a short lactation period, treatment with amphotericin B or itraconazole can be recommended; however, in severe cases with large dissemination, the drug of choice is lipidic amphotericin B (3–5 mg/kg/day) or the deoxycholate form (0.7–1 mg/kg/day) which can be complemented with local hyperthermia [88]. Cryosurgery is another safe and successful alternative [95].

### 6.4. Sporotrichosis in Children

Pediatric sporotrichosis is rare in Latin American countries and has been poorly analyzed. The most common clinical forms of sporotrichosis in this studied population is the lymphocutaneous form followed by the fixed cutaneous form [96,97].

In 2015, Ramirez Soto et al. reviewed 1503 cases of sporotrichosis in a low-socio-economic area of Peru (Abancay), in which children ≤14 years old accounted for 62% of the sporotrichosis cases [98]. In a subsequent retrospective study of sporotrichosis in 240 pediatric patients of the same region, data showed that 54.6% were males and the median age for acquired sporotrichosis was 6 years (4–10) [99]. Data showed that the incidence of lymphocutaneous sporotrichosis and fixed sporotrichosis was 55 and 27 cases per 100,000 person-years, respectively. The face was the most usually affected site, and 15 cases showed sporotrichosis in the ocular adnexa; 40% of the infected patients reported previous contact with a sick cat and 19.2% of them reported a history of traumatic inoculation with plant material.

Disseminated cutaneous sporotrichosis caused by *S. brasiliensis* with sepsis of a cutaneous focus was described in a young immunocompetent adolescent from Brazil [99].

Therapy in children included amphotericin B (0.7 mg/kg/day) and/or itraconazole (6-10 mg/kg/day) or potassium iodide (two oral drops three times daily at the beginning of the treatment, increasing the dose by two drops at each session to a maximum of 20 drops three times daily) depending on the clinical form [85]. Among cutaneous-osteoarticular cases, itraconazole and/or potassium iodide combined with sulfamethoxazole/trimethoprim (800 mg/160 mg) twice daily rendered good results [100].

### 6.5. Sporotrichosis in Cats

Cats can be infected with *S. brasiliensis* almost exclusively through the enzootic route via traumatic inoculation of the fungus through bites and scratches from infected cats or by a non-traumatic route through direct contact with exudates from sick cats. Coughs and sneezing are believed to be potentially transmitters of feline sporotrichosis [101]. However, the sapronotic route of transmission should not be discarded, as it is a common route of transmission for other *Sporothrix* species present in soil, organic matter or decaying plants, via their traumatic inoculation. Sporotrichosis has been mainly reported in free-roaming intact male cats [102].

Sick cats are considered the main source of fungal transmission due to the high yeast burden in their lesions. *S. brasiliensis* was isolated from the small intestinal contents of necropsied cats as well as from cat feces collected from sand in Sao Paulo. Feces from infected cats may contaminate the soil, creating an environmental reservoir for *S. brasiliensis* and a possible new source of contamination for animals and humans [103]. Something similar may occur with the buried cat. In the zoonotic cycle of sporotrichosis, the feline claws can be the first acquisition site of *Sporothrix* propagules when digging in soil or sharpening on the bark of a tree. During licking, propagules might direct to its mouth/nose [19].

Strikingly, sporotrichosis due to *S. brasiliensis* can also be transmitted from “healthy” or asymptomatic cats during the incubation period. In this regard, nail cultures from healthy cats were positive in 0.57% of them in Brazil [40]. Therefore, asymptomatic carriage and transmission of *S. brasiliensis* from healthy cats is possible and may constitute an underestimated problem. In Argentina, our research group in Medical Mycology failed to isolate *Sporothrix* spp. from the oral/nasal cavity and claws of 33 healthy young cats, from the locality of Tigre, Buenos Aires province, which is one of the urban areas where CTS and feline sporotrichosis have been recorded.

Cats with sporotrichosis often have lesions on their faces, mostly around the nose, and in their limbs. These lesions develop from wounds that occur during fights with an infected cat. Cats that lick infected wounds on other parts of their bodies can also transfer the fungi to their faces and mouths. The most common manifestations of feline sporotrichosis are ulcerated lesions on the skin (deep wounds) that do not heal and tend to evolve quickly. Respiratory signs such as sneezing have also been reported. This disease is typically more serious in cats than in humans and frequently evolves to the systemic disseminated form, which is often fatal [3].

In contrast to humans, severe feline sporotrichosis may develop independently of retrovirus coinfections (*Feline immunodeficiency virus*, FIV or *Feline leukemia virus*, FeLV), which are known to cause immunosuppression in cats (high IL-10, and low IL-4 and IL-12 levels) and thus influence their clinical presentation as in HIV-co-infected humans [103]. In this regard, a study conducted by Miranda et al. showed that all cats that had the most severe clinical presentation and poor general condition were co-infected with FeLV or FIV [104]. In most of the endemic areas of Rio de Janeiro, as well as in the Argentinean urban areas where feline sporotrichosis cases were documented, cats are usually allowed to roam outdoors, and most of them are neither vaccinated nor neutered, not receiving regular prophylactic deworming. Therefore, the population of stray cats is large, and the contribution of other infectious diseases (i.e., tapeworms, retroviruses) to immunosuppression and hence to the susceptibility of cats to sporotrichosis cannot be ruled out. In this regard, Maizels et al. described that among parasites, helminths are potent inducers of immune anergy and anti-inflammatory responses as well as important agents that drive a Th2-biased immune response [105]. In addition, helminth and fungus concomitant infections are common in developing countries. In Argentina, rats are important sources of the cestode *Taenia taeniaformis* [106] and according to Zhang et al., this parasitic co-infection among rats with *S. schenckii* elicit conflicting immune pathways that counterbalance each other inducing changes in the cytokine profile and reducing the host´s ability to clear this fungal infection [107]. A similar situation could not be ruled out in cats with sporotrichosis co-infected with *T. taeniaformis*. Therefore, it is important that cats receive prophylactic deworming.

With regard to retroviruses, especially FIV, which exhibits a marked cellular tropism towards CD4+ cells, a major contribution is expected in the presentation and manifestation of sporotrichosis (as occurs in cryptococcosis, where the alteration in the CD4 / CD8 ratio, fundamentally observed in cats with FIV, favors the dissemination of the fungal agent). However, due to the chronic course of the disease caused by the viral strains circulating in Latin America, it might take many years for the CD4/CD8 ratio to be altered, thus favoring the clinical manifestation of the fungus. As in sporotrichosis, FIV can be transmitted to other susceptible cats by territorial disputes that may lead to bite wounds, in which the infected cat saliva enters the other cat tissues. Thus, it is likely that during a fight, coinfection with both infectious agents (FIV and *S. brasiliensis*) occurs at the same time. Nevertheless, the chronic course of the FIV disease that leads to the alteration of the CD4/CD8 ratio, could attempt against the clinical manifestation of severe sporotrichosis in cats with FIV.

On the other hand, not all cats are accompanied by their owners (in the case of FIV) throughout the disease. Many of these cats are euthanized before reaching the final stages (for various reasons, including financial ones). Therefore, it is difficult in many countries to individualize the group of cats at the final stage of FIV to further evaluate the presence of *Sporothrix*.

Although FeLV has also tropism towards the lymphocyte line, the greatest depletion in CD4 cells and the reduction in the CD4/CD8 ratio is not as sharp as it is in FIV, hence, a severe course of the fungal disease is not as expected as in VIF. However, the greatest limitation in FeLV is found in the diagnosis. In many cases, the diagnosis of this retrovirus must be made by complementing different techniques such as immunochromatography and polymerase chain reaction (PCR). Many cats have the viral disease and are not diagnosed. Moreover, there are others who acquire it and immediately die due to the virulence of the virus and its effects, without the manifestation of the fungal disease.

Hence, the possibility of association between cats co-infected with retroviruses and *Sporothrix* should not be ruled out. Further investigations should be conducted taking into account these considerations to further conclude whether or not such an association exists. Until this occurs, cats should not have free access to the streets, overcrowding should be avoided, and all types of sources of contagion should be minimized. Finally, cats with symptoms of sporotrichosis should be routinely tested for retroviruses and the possible benefits of antiviral therapy in patients with both diseases and refractory to mycological treatment should be evaluated to avoid aggravating the antifungal resistance issue in *S. brasiliensis.* With respect to the diagnosis, feline sporotrichosis should be included in the differential diagnosis of nodular and/or ulcerative skin disease with draining tracts, especially in cats in which a bacterial infection was initially suspected but the response to antibiotics was poor and/or in cats that live or come from an endemic area for sporotrichosis [102].

Although the treatment of feline sporotrichosis is shown to efficiently reduce the fungal burden in lesions of cats contributing to the control of zoonotic transmission, it still remains a challenge for veterinarians. Itraconazole capsules at 5–10 mg/kg/day frequently in association with potassium iodide capsules at 2.3–20 mg/kg/day are still the treatment of choice, although therapeutic failure, adverse effects and relapse of lesions were described. Additionally, antifungal therapy may take too long (up to nine months), and therapy maintenance for one month after clinical remission is highly recommended. However, in severe cases, it is recommended for two months after clinical cure to minimize the risk of recurrence [1], which is usually frequent in feline sporotrichosis. Therefore, compliance with the therapeutic protocol by the cat owner is crucial to achieve a therapeutic success in the sick pet. It was reported that the occurrence of respiratory signs, nasal mucosa lesions and skin lesions with high fungal burden and fungus-rich granulomas are associated with treatment failure and death [3,108].

Amphotericin B, terbinafine, ketoconazole, local heat treatment, adjunctive surgical treatment, and cryosurgery with or without concurrent itraconazole are other less common options for the treatment of feline sporotrichosis [3].

### 6.6. Sporotrichosis in Dogs

Cases of canine sporotrichosis have rarely been reported. However, during the period 2004-2014 in Brazil, >200 canine cases of sporotrichosis due to *S. brasiliensis* were identified [109], most of which were related to CTS [110]. However, dogs are not reported as a source for human infections of *S. brasiliensis*, and no cases of dog-transmitted sporotrichosis to human have been reported so far with this fungal species. In contraposition with sick cats, ill dogs carry a low burden of yeast cells in their lesions [109].

The cutaneous and lymphocutaneous forms of sporotrichosis with ulcers and/or nodules on the head, nose, ears, neck, chest and limbs are the most common clinical forms described in dogs. Extracutaneous signs, in particular, respiratory signs such as sneezing, dyspnea and nasal discharge might also occur. Osteoarticular and disseminated forms were also reported [111].

For the therapy of canine sporotrichosis, oral itraconazole, ketoconazole and potassium iodide are the most common drugs used. Oral terbinafine (25–30 mg/kg/day) might also be used [112].

## 7. Implementation of Novel Tools to Improve the Diagnosis of Sporotrichosis

Sporotrichosis goes mostly undiagnosed and is not even on the diagnostic algorithm used by a significant proportion of clinicians facing a sick cat/patient in the site exhibiting cutaneous “sporotrichoid” lesions. The panorama is even bleaker in cases with the ocular, immunoreactive and extracellular forms of the disease, which are even more difficult to recognize clinically and, thus, the diagnosis is a real challenge.

The absence of a simple, reliable, and affordable diagnostic test has made it difficult to determine the burden of this disease in many endemic countries for sporotrichosis. The gold standard for diagnosis relies, so far, on the culture of fluids/secretions and tissue samples [11]. However, it is time consuming (5–8 days) and its sensitivity might be low. In this regard, it was reported that in patients with both HIV and sporotrichosis, *S. brasiliensis* can be isolated from cerebrospinal fluid (CSF) in a low percentage (14.3%) [91]. Demonstration of dimorphism is needed to confirm the suspected fungal agent.

From the lab perspective, direct examination of fungal elements in the lesions may accelerate diagnosis, at least in cats due to their high fungal load. However, its sensitivity is low for the diagnosis of human sporotrichosis (<20%) due to the scarcity of fungal elements in their lesions and yeast cells are usually non-specific. In addition, none of the aforementioned techniques is able to distinguish *Sporothrix* isolates to the species level. For this purpose, phenetic (morphology of the pigmented sessile conidia), physiological tests (sugar assimilation assay and growth at different temperatures: 20 °C, 30 °C, 35 °C and 37 °C) and molecular analyses (mainly based on DNA amplification of the spanning exons 3´ to 5´ of the calmodulin (CAL) gene, followed by DNA sequencing or digestion of the amplicon with the restriction enzyme *Hha*I by RFLP) are used [9,51]. More recently, a multiplex real-time PCR method based on the CAL gene for the identification of the clinically relevant *Sporothrix* species: *S. globosa, S. schenckii sensu stricto* and *S. brasiliensis* was developed, rendering good results [113]. Detection of specific antigens in serum or urine samples is not yet available for diagnosis, and antibody detection by enzyme-linked immunosorbent assay (ELISA) that can differentiate acute disease from past infection is not being used as a standard of care. Most often, clinicians and particularly, veterinarians do not take proper samples for mycological diagnosis, thus perpetuating the absence of a reliable diagnosis and, consequently, the continuous “non-existence” of this endemic disease.

Among the identification markers currently used for the molecular identification of *S. brasiliensis,* the nuclear rDNA internal transcribed spacer region (ITS-5.8S-ITS2), CAL, β-tubulin (BT2) and the elongation factor 1α (EF-1α) are included. The former is the official DNA barcode for fungi [114] although sometimes it does not contain enough variability for distinguishing among all *Sporothrix* species. The remaining ones are used as a secondary barcode or identification marker, which is usually needed to reliably identify a *Sporothrix* culture at the species level. Of those, CAL is the most widely used because, as it is easy to amplify, distinguishes among all human pathogenic *Sporotrhix* species, and even rare agents such as *Sporothrix pallida, S. mexicana* and *Sporothrix chilensis*. In addition, the CAL sequence database is almost complete for all accepted species.

Concerning the serological diagnosis, a latex agglutination (LA) test is commercially available (LA-*Sporothrix* Antibody System^TM^, Immuno Mycologics Inc.) for the qualitative detection of agglutinating antibodies in serum samples from patients with sporotrichosis; however, its sensitivity depends on the clinical form: 56% for cutaneous forms, 73% for pulmonary sporotrichosis, 86% for osteoarticular forms and 100% for disseminated sporotrichosis [1].

At present, few antigens have been used as diagnostic reagents. Among them, the antigenic purified PRM fraction denominated *S. schenckii* concanavalin A binding fraction (SsCBF) and a *S. schenckii* crude exoantigen preparation were used to develop an ELISA test for the serodiagnosis of human and feline sporotrichosis. SsCBF is a purified antigen obtained by fractioning the cell wall PRM by an affinity chromatography on Sepharose 4B-Concanavalin A. While in this antigen, one epitope is the target for antibody recognition, in the crude exoantigen, multiple epitopes are involved in the antibody detection [115]. The SsCBF-based immunoassay was clinically validated for cat and human sporotrichosis with high sensitivity and specificity and is currently used as an “in-house” test in two University Hospitals in Rio Janeiro, Brazil [31]. This ELISA is also useful for therapeutic monitoring [116]. In a study conducted by Ferreira Fernandes et al., using sera from 30 cats with proven sporotrichosis, the SsCBF ELISA showed 90% sensitivity and 96% specificity, whereas the ELISA coated with the crude exoantigen preparation rendered 96% sensitivity and 98% specificity [117].

A lateral flow assay for the diagnosis of CTS was recently developed using the SsCBF antigen being currently under evaluation and validation in Brazil using serum samples of different clinical forms of sporotrichosis. Rodrigues et al. also proposed 3-carboxymuconate cyclase as a potential diagnostic and vaccine antigen target. One-dimensional immunoblotting showed that this protein is the immunodominant antigen in feline sporotrichosis whereas two-dimensional immunoblotting showed six immunoglobulin (IgG)-reactive isoforms of this antigen in the *S. brasiliensis* proteome, similar to the humoral response found in human sporotrichosis [55].

More recently, proteomic profiles generated by matrix-assisted laser desorption/ionization time-of-flight mass spectrometry (MALDI-TOF MS) have been used for fungal identification as a reliable, fast and cost-effective method [115]. Since an accurate database is essential and it is not available for all medically important fungal species, such as *Sporothrix* spp., MALDI-TOF MS associated with an in-house database enriched with reference *Sporothrix* complex spectra was described for the identification of *S. brasiliensis* [81,118].

The antifungal susceptibility test and minimum inhibitory concentration (MIC) values for the filamentous and yeast forms of *S. brasiliensis* against different antifungal drugs were described using the M38-A2 and M27-A3 Clinical and Laboratory Standards Institute CLSI guidelines, respectively [119]. Recently, Almeida-Paes et al. studied the MIC distributions of five antifungal drugs (amphotericin B, itraconazole, ketoconazole, posaconazole and terbinafine) against the filamentous form of *S. brasiliensis* according to the M38-A2 CLSI guidelines and proposed the first tentative epidemiological cutoff values (ECVs, 4.0, 2.0, 1.0, 2.0 and 0.25 µg/mL, respectively) [120]. However, a more recently multicenter, international study conducted by Espinel-Ingroff et al. proposed the following ECVs for the filamentous form using the CLSI M38-A2 document: amphotericin B 4.0 µg/mL, itraconazole 2.0 µg/mL, posaconazole 2.0 µg/mL, voriconazole 32 µg/mL, ketoconazole 2.0 µg/mL and terbinafine 0.12 µg/mL [121]. These findings indicate that terbinafine has the highest in vitro inhibitory activity, whereas voriconazole the lowest against *S. brasiliensis*. It should be pointed out that although ECV is not a predictor of clinical response to antifungal therapy, MIC values higher than the proposed ECV may suggest that these species are non-wild type and potentially refractory to antifungal therapy, but not necessarily resistant or unresponsive to therapy. Similarly, MIC values lower than ECV do not necessarily indicate success or responsiveness to antifungal therapy. Indeed, cases of refractory sporotrichosis due apparently to wild-type isolates of *S. brasiliensis* in humans were reported [122].

## 8. Exploring New Antifungal Agents

As mentioned above, the current first-line therapy for feline and human sporotrichosis is itraconazole, although potassium iodide and terbinafine can be used as therapeutic alternatives for the cutaneous forms of the disease, and amphotericin B for the most severe cases (extracutaneous and disseminated). This poor drug armamentarium together with therapeutic failures, side effects and emergence of drug-insensitive isolates highlights the importance of searching for novel therapeutic strategies. Recent genome data and bioinformatic analyses have helped to identify specific fungal enzymes, such as those involved in the biosynthetic glycosylation routes, in the synthesis of the fungal cell wall or in the synthesis of ergosterol (other than at the C14α-demethylase stage), as putative targets for the development of novel antifungal drugs. In this regard, particular attention was given to inhibitors of Δ^24^-sterol methyltransferase (24-SMT, an enzyme of ergosterol biosynthesis restricted to plants, protozoa and fungi), such as 22-hydrazone-imidazolin-2-yl-chol-5-ene-3β-ol (H3) which completely depleted ergosterol in *S. schenckii* and *S. brasiliensis*, in contrast to itraconazole that partially blocked its synthesis. However, in combination with itraconazole, H3 enhanced the effectiveness of the triazolic compound. In addition, cytotoxicity assays showed that H3 was more selective towards these fungi than itraconazole. All these results suggest that 24-SMT is a promising target for novel antifungal therapies against sporotrichosis, either as sole treatments or in combination with itraconazole [123].

However, since the discovery of novel, effective and stable antifungals is an expensive long-term process, few synthetic compounds, natural products and crude extracts have been investigated and are discussed below.

Asquith et al. developed a library of pentathiepin-based inhibitors of *S. brasiliensis*, where some of them (the 2,5-dichloro, N-methyl and the 2,5-dichloro, N-isopropyl compounds) were more potent than itraconazole [124]. Pentathiepins are high-density polysulfide heterocycles whose pentathiepin functional motif is not toxic and has the potential to generate a novel class of antifungal drugs for the therapy of sporotrichosis in cats and eventually in humans [124].

Several synthetic substitutes α- and β-2,3-dihydrofuranophotoquinones were also shown to be inhibitors of fungal growth. Their antifungal activity against *S. brasiliensis* was described to be exerted by inducing yeast-hypha conversion and alteration in the hyphae and conidia structures [125].

The organoselenium compound diphenyl diselenide (PhSe)_2_ also displayed antifungal activity against *S. brasiliensis* either alone and in combination with itraconazole due to its pro-oxidant effects on fungal cells with possible inhibition of melanin synthesis and an anti-oxidant effect in mammalian cells [126]. Hence, PhSe_2_ might have a beneficial effect on the host and a detrimental effect on the fungus. It should be noted that melanin inhibitors are widely used as agrichemical fungicides. In this regard, Almeida-Paes et al. reported that in the presence of the DHN-melanin inhibitor tricyclazole, the in vitro susceptibility of *S. brasiliensis* against terbinafine increased, demonstrating that melanin protects this fungus from the effects of terbinafine and that the development of new antifungal drugs targeting melanin synthesis can improve sporotrichosis treatments [68]. The inhibitors sulcotrione and glyphosate also reduce the MIC values of *S. brasiliensis* but in a minor proportion compared to tricyclazole [68].

Gagini et al. have shown that the imidazolic drug clotrimazole is effective *in vitro* against *S. brasiliensis* strains, and that is more active against feline isolates than itraconazole [127]. Moreover, in combination assays, clotrimazole increased the antifungal activity of itraconazole. However, human isolates were more sensitive to itraconazole. It should be noted that feline and human isolates of *S. brasiliensis* had similar susceptibility profiles to itraconazole [126]. The strong antifungal activity against feline isolates suggests that the use of topical formulations of clotrimazole alone or in combination with oral itraconazole represents a potential alternative for the topical therapy of feline sporotrichosis [127].

A microemulsion formulation containing both azoles at the same concentration (1%) was then developed as a transdermal delivery system for the treatment of sporotrichosis. This formulation composed of benzyl alcohol (oil phase), Tween^TM^ 60 (surfactant) and the transdermal permeation promoter propylenglycol or Transcutol^TM^ (cosurfactant) has the possibility to achieve a systemic delivery alternative to the oral route [128]. Ferreira et al. also demonstrated a dual activity of clotrimazole in this transdermal formulation: as a promoter enhancer and as an active antifungal compound, which in association with itraconazole constitutes a novel therapeutic tool for the transdermal treatment of sporotrichosis. 

The antifungal activity of hydroalcoholic extracts of brown Brazilian propolis (*Api mellifera*) was also proven against *S. brasiliensis* (including itraconazole-resistant isolates) representing a new antifungal candidate for the treatment of animal sporotrichosis, including against itraconazole-resistant isolates [129]. Essential oils and polar extracts of rosemary (*Rosmarinus officinalis* L.), oregano (*Origanum vulgare* L.) and marjoram (*Origanum majorana* L.) have also shown anti-*S. brasiliensis* fungistatic and/or fungicidal activity depending on the concentration [130,131].

Garcia et al. assessed the antifungal activity of different molecular weight chitosans against planktonic cells and biofilms of *S. brasiliensis* [132], where low molecular weight chitosan showed the best inhibitory activity against both planktonic cells and biofilms. However, all three molecular weight chitosans demonstrated activity against both planktonic cells and biofilms of *S. brasiliensis*.

Brilhante et al. evaluated the effects of exposure to amphotericin B, itraconazole, caspofungin, ketoconazole, voriconazole and fluconazole against the planktonic form on the biomass and metabolic activity of these biofilms, and the best inhibitory response was observed with amphotericin B and caspofungin, probably because they damaged the architecture and extracellular matrix, allowing diffusion of the drugs [53].

Nikkomycin Z, a uridine-based nucleoside-peptide antibiotic that inhibits fungal chitin biosynthesis by blocking chitin synthase, showed an in vitro inhibitory activity against *S. brasiliensis*. In combination with itraconazole, nikkomycin Z exhibited a synergistic antifungal effect [133]. Similarly, Borba-Santos et al. reported that the calcineurin inhibitors tacrolimus and cyclosporin A alone inhibited the yeast growth of *S. brasiliensis*. However, only tacrolimus exerted a synergistic effect when combined with itraconazole or fluconazole [134]. This effect might be related to the inhibition of ABC efflux transporters by tacrolimus.

As mentioned above, amphotericin B deoxycholate (D-AMB) or its lipid formulations and/or in association with itraconazole are used in severe cases of sporotrichosis. Nephrotoxicity is the major side effect of D-AMB; however, the best security profile exhibited by the lipid formulations are hampered by higher treatment costs, thus preventing their widespread use, particularly in developing countries, where sporotrichosis is endemic. Ishida et al. prepared a non-lipid formulation of a poly-aggregated amphotericin B (P-AMB) and demonstrated its in vitro and in vivo efficacy in a systemic model of sporotrichosis due to *S. brasiliensis*, with a minor cytotoxic effect on renal cells and erythrocytes compared to D-AMB. Therefore, P-AMB could be considered an alternative drug to D-AMB for the treatment of systemic sporotrichosis [135].

A humanized monoclonal antibody against a 60–70 kDa glycoprotein (gp60-70) of the cell wall of *S. schenckii*, and *S. brasiliensis* (P6E7) was developed by Almeida et al. as a potential therapeutic and prophylactic tool against sporotrichosis. Opsonization of yeasts, followed by an increase in the phagocytic index of monocyte-derived macrophages and reduction of disease burden was observed in an in vivo model of sporotrichosis [136].

## 9. Exploring Vaccine Candidates

At present, there is no effective vaccine available to prevent sporotrichosis. Few antigens were identified as putative vaccines candidates for the effective control of *Sporothrix* infection.

Portuondo et al. studied the efficacy and toxicity of two vaccine formulations using *S. schenckii* cell wall proteins (ssCWP) as immunogens, with Montanide^TM^ Pet Gel A (PGA) or aluminum hydroxide (AH) as adyuvants in mice [137]. The ssCWP contains at least four antigenic molecules recognized by sera of immunized mice: a 44kDa peptide hydrolase, a 47kDa enolase, which was predicted to be an adhesin, a 71 kDa protein and a 9.4 kDa peptide [57]. Results showed that the PGA formulation was able to confer the same level of protection as aluminum hydroxide in immunized mice with the advantage of only minimal local reactions, making it a potential alternative as a future veterinary vaccine against sporotrichosis by *S. brasiliensis* due to it cross-reactivity [137]. Furthermore, Portuondo et al., developed a PGA-adjuvanted vaccine formulation using recombinant enolase obtained in *Escherichia coli* BL21 clones and demonstrated that it was able to confer protection either actively to a subsequent challenge with *S. brasiliensis* or passively through the serum of vaccinated mice. Therefore, enolase could be used as a potential antigenic target for vaccinal purposes against sporotrichosis [57].

Other potential *Sporotrhix* vaccine targets include a 100 kDa antigenic molecule (an endoplasmic signal peptidase), the peptide hydrolase (44 kDa), the 3-carboxymuconate cyclase (gp60-70), and the ZR8 peptide from the GP60-70 protein, which is the main antigen of the *Sporothrix* complex and was shown to be the best potential vaccine candidate by increasing CD4+ T cells and higher levels of FN-Ɣ, IL-17A, and IL-1β, which characterize a strong cellular immune response with induction of a high number of neutrophils in the lesions that are associated with fungus clearance [2,55,138].

## 10. Conclusions

The concept of “One Health” was introduced in the early 2000s as a global approach that recognizes that human and animal health are interdependent and linked to the health of the ecosystems in which they live. This approach to health is a shared effort of many professionals with expertise and activity in different sectors that join forces in order to detect, respond and prevent zoonosis outbreaks. To achieve this purpose, laboratory information and epidemiological data must be shared among different sectors; and sanitary authorities, veterinarians, and healthcare workers must implement effective strategies at global, regional and local levels against health threats. 

Unfortunately, *S. brasiliensis* is still causing widespread infections in Brazil. However, it is now expanding into neighboring countries such as Argentina. The Brazilian experience warns that once settled in a community, these infections may spread much further. Given the travel and exposure patterns of humans and cats, physicians and veterinarians need to be prepared to recognize and treat infections caused by *S. brasiliensis* in other geographical regions.

The collaboration between all these health-care workers and sanitary authorities would be critical in facilitating the detection of epidemics early on and preventing further dissemination. 

It is quite explicit that collaborative, multisectoral and transdisciplinary research networks are necessary to address four great challenges related to this emerging fungus [139]: (i) to gain a deeper insight into the forces driving its emergence, evolution, and spread; (ii) to recognize the different mechanisms of fungal adaptation and interaction with hosts and the environment; (iii) to understand the evolution of resistance to antifungals among other members of the *S. schenckii* complex; and (iv) to implement current successful diagnostic approaches and develop novel strategies to control and prevent this fungal disease. Human activity, social behaviors, together with environmental and climate changes (i.e., global warming), intensify the impact of emerging fungi such as *S. brasiliensis.* We need to stop forgetting and underestimating fungal diseases and GAFFI is the voice of this claim by considering sporotrichosis a high priority mycosis. We need efforts to reduce illness and death associated with this fungal disease, and to improve public awareness and clinical expertise, by supporting education, promoting advocacy and developing training programs.

It is widely known that the negative impact of any infectious disease is frequently higher in neglected communities. Delayed diagnoses, lack of access to healthcare systems and antifungal therapy, for instance, lead to a higher burden of fungal diseases. Recently, the World Health Organization launched a new road map for neglected tropical diseases (NTD) for 2021–2030 and included sporotrichosis as one of the unspecified deep mycoses recognized as fungal NTDs [140].

Further research is needed and there is still a long way to go to better understand several key aspects (i.e., fungal biology, pathogenesis, urinary and serum biomarkers) of this emerging fungus. The phrase “know your enemy” fits well in this scenario. We need simple, reliable, and affordable diagnostic tests, therapies and vaccines; however, first of all, physicians and veterinarians need to integrate the South American epidemiology into their diagnostic algorithms. For instance, suspecting leishmaniasis in patients with “sporotrichoid” lesions from endemic areas is not an issue; suspecting sporotrichosis in cats or in immunosuppressed, HIV-infected patients in South America, and perhaps other geographical regions, is.

With one fungus detected, the “One Health” approach is promoted.

## Figures and Tables

**Table 1:** Human cases of sporothrichosis caused by *S. brasiliensis* in Argentina.

Year	Locality (Province)	Sex	Age (Years)	Source of Infection (Connection)	Clinical Form	Diagnosis
1986	N.A. (Misiones)	N.A.	N.A.	N.A.	N.A.	Culture and Molecular
1988	N.A. (BA)	N.A.	N.A.	Cat	N.A.	Culture and Molecular
1990	N.A. (BA)	Female	8	Cat (Cat owner)	LC	Culture
2011	N.A. (BA)	N.A.	N.A.	Rat	N.A.	Culture and Molecular
2011	Paso del Rey (BA)	Male	67	Cat (Cat owner)	LC	Culture and Molecular
2011	Paso del Rey (BA)	Female	67	Cat (Cat owner)	FC	Culture and Molecular
2012	San Miguel (BA)	Male	35	Cat (Cat owner)	LC	Culture and Molecular
2012	Moreno (BA)	Female	50	Cat (Cat owner)	LC	Culture and Molecular
2016	Malvinas Argentinas (BA)	Female	61	Cat (Cat owner)	LC	Culture and Molecular
2017	N.A. (BA)	N.A.	N.A.	N.A.	N.A.	Culture and Molecular
2018	Los Polvorines (BA)	Female	3	Cat (Cat owner)	FC	Culture and Molecular
2018	Los Polvorines (BA)	Female	33	Cat (Veterinarian)	LC	Culture and Molecular
2019	Tigre (BA)	Female	30	Cat (Cat owner)	FC	Culture and Molecular
2019	Tigre (BA)	Female	37	Cat (Veterinarian)	FC	Culture and Molecular
2019	El Calafate (SC)	N.A.	N.A.	Cat (Catowner)	N.A.	Culture and Molecular
2019	El Calafate (SC)	N.A.	N.A.	Cat (Cat owner)	N.A.	Culture and Molecular
2019	El Calafate (SC)	N.A.	N.A.	Cat (Cat owner)	N.A.	Histopathology
2019	El Calafate (SC)	Female	32	Cat (Veterinarian)	N.A.	Histopathology
2019	N.A. (BA)	Male	N.A.	Cat	LC	Histopathology
2019	N.A. (BA)	Female	4	Cat (Cat owner)	LC	Culture
2019	Moreno (BA)	Male	22	Wood splinter (Workshop worker)	LC	Culture and Molecular

N.A.: Not available, BA: Buenos Aires, SC: Santa Cruz, LC: lymphocutaneous, FC: fixed cutaneous.

**Table 2:** Feline cases of sporotrichosis caused by *S. brasiliensis* in Argentina.

Year	Locality/Province	Sex	Age (Years)	Breed	Reproductive Status	Access to Street	FIV/ FELV	Clinical Presentation	Outcome	Zoonotic Transmission to Humans	Diagnosis
1988	N.A.	N.A.	N.A.	UB	N.A.	Yes	N.A.	Localized	N.A.	Yes	Culture and Molecular
2006	Tigre/BA	Male	1.5	UB	Entire	Yes	N.A.	Disseminated	N.A.	N.A.	Culture
2008	Paso del Rey/BA	Male	4	Siamese	Entire	Yes	N/N	Localized	Recovery after therapy with itraconazole	No	Culture
2011	Paso del Rey/BA	Male	2	UB	Entire	Yes	N/N	Localized	Recovery after therapy with itraconazole	Yes	Culture
2012	San Miguel/BA	Female	2	UB	Entire	Yes	N.A.	Localized	Recovery after therapy with itraconazole	Yes	Cytology
	San Miguel/BA	Female	2	UB	Entire	Yes	N.A.	Disseminated	Euthanasia	Yes	Cytology
	Moreno/BA	Male	2	UB	Entire	Yes	N.A.	Localized	N.A.	Yes	Culture and Molecular
	Paso del Rey/BA	Female	2	UB	Entire	Yes	N.A.	Localized	N.A.	No	Cytology
2013	Florida/BA	Male	2	UB	Neutered	Yes	N/N	Disseminated	Death	No	Culture
	Grand Burgh/BA	Female	2	UB	Neutered	Yes	N/N	Localized	Recovery after therapy with itraconazole	No	Culture
2016	San Isidro/BA	Female	N.A.	N.A.	N.A.	N.A.	N.A.	N.A.	N.A.	N.A.	N.A.
	San Isidro/BA	Female	2	UB	N.A.	N.A.	N.A.	N.A.	Recovery after therapy with terbinafine	N.A.	Cytology
	Mar del Plata/BA	Female	2	Siamese	Neutered	Yes	N/N	Localized	Recovery after therapy with itraconazole	No	Cytology
	Los Polvorines/BA	Male	10	UB	N.A.	N.A.	N.A.	Disseminated	Death	Yes	Epidemiology and clinic
2017	Tigre/BA	Female	2	UB	Neutered	Yes	N.A.	Localized	Recovery after therapy with itraconazole	No	Culture and Molecular
	Tigre/BA	Female	2	UB	Neutered	Yes	N.A.	Localized	Recovery after therapy with itraconazole	No	Culture and Molecular
2018	Tres de Febrero/BA	Female	2	UB	Castrated	Yes	N/N	Localized	Recovery after therapy with itraconazole	Yes	Culture and Molecular
2019	Tres de Febrero/BA	Male	4	UB	Neutered	Yes	P/N	Disseminated	Recovery after therapy with itraconazole	No	Culture and Molecular
	Tigre/BA	Male	2	UB	Neutered	Yes	N.A.	Disseminated	Recovery after therapy with itraconazole	Yes	Culture and Molecular
	Ciudad Evita/BA	Male	N.A.	UB	Entire	Yes	N.A.	Localized	N.A.	Yes	Culture and Molecular
	El Calafate/SC	Male	N.A.	UB	N.A.	N.A.	N.A.	Disseminated	Death	Yes	Histopathology
	El Calafate/SC	Male	N.A.	UB	N.A.	N.A.	N.A.	Disseminated	Death	Yes	Histopathology
	El Calafate/SC	Male	N.A.	UB	N.A.	N.A.	N.A.	Disseminated	Death	Yes	Histopathology
	El Calafate/SC	Male	N.A.	UB	N.A.	N.A.	N.A.	Disseminated	Death	Yes	Histopathology
	El Calafate/SC	Male	N.A.	UB	N.A.	N.A.	N.A.	N.A.	N.A.	No	N.A.
	El Calafate/SC	Male	N.A.	UB	N.A.	N.A.	N.A.	N.A.	N.A.	No	N.A.

BA: Buenos Aires, SC: Santa Cruz, N.A.: not available, UB: undefined breed, N/N: negative/negative, P/N: positive/negative.

**Table 3:** Virulence factors of *S. brasiliensis.*

Virulence Factors	Function	Cellular Location
Thermal dimorphism	Establishment of the infection	-
Thermotolerance	Survival and parasitism	-
Biofilm	Drug resistance	Extracellular
Extracellular cell wall glucanase	Immune evasion and secretion of toxic factors	Extracellular
Extracellular vesicles	Drug resistance, cell invasion and pathogenesis	Extracellular
3-carboxymuconate cyclase	Adhesion to extracellular matrix	Cell wall
Enolase (2-phospho-d-glycerate hydrolase)	Adhesin	Cell wall
Cell wall lipids	Phagocytosis inhibition	Cell wall
Melanin	Drug resistance, protection against nitrogen-derived oxidants; phagocytosis resistance; dissemination of internal organ phagocytosis. Penetration into tissues and evasion from immune system	Cell wall
Glyceraldehyde-3-phosphate dehydrogenase	Glycolysis and adhesin	Cytosol
Progesterone binding protein	Dimorphism	Cytosol
Rhamnolipid biosynthesis 3-oxacyl-(acyl-carrier-protein) reductase	Immune modulation, antimicrobial activity, biofilm development, surface motility	Cytosol
Hydroxymethylglutaryl-coenzyme A lyase	Immune evasion	Cytosol, endoplasmic reticulum
Heat shock 70-kDa protein 1/8	Immune evasion and secretion of toxic factors	Cytosol, mitochondria, plasma membrane, endoplasmic reticulum and nucleus
Mn superoxide dismutase	Immune evasion and secretion of toxic factors	Mitochondria
Acetyl-coenzyme A hydrolase	Carbohydrate, lipid and protein metabolism	Mitochondria
Aminopeptidase I	Immune evasion and secretion of toxic factors	Vacuole
Urease	promotes penetration into tissues and evasion from the immune system	Vacuole

**Table 4:** Clinical forms of sporotrichosis.

Clinical Form	Main Characteristics
Cutaneous	Usually appears after a minor trauma that alters the integrity of the epidermis.
Fixed	Yeasts remain localized in the subcutaneous tissue. One or few lesions occur at the inoculation site, which are often ulcerated with erythematous edges. Lesions may be ulcerated, verrucous, plaque infiltrated, or tuberous. Without lymphatic involvement.
Lymphocutaneous	Yeasts spread to adjacent lymphatic vessels. The primary lesion is usually located on the extremities, especially hands and forearms. The lesion has a papulonodular appearance and may ulcerate and fistulize. Secondary lesions arise along the regional lymphatic channels. Lymph node involvement or systemic symptoms are unusual. The most frequent clinical form (>75% affected patients).
Cutaneous-disseminated	Yeasts spread by the hematogenous route. Multiple skin lesions occur at noncontiguous sites. Without extracutaneous involvement. Mainly seen in immunocompromised hosts.
Mucosal	May be caused by self-inoculation through fungus-contaminated hands, hematogenous dissemination or conidia inhalation. Preauricular and submandibular lymph node enlargement is frequent.
Nasal	Lesions often involve the septum, producing bloody secretions and detachment of crusts.
Ocular	May be produced by hematogenous spread or fungal inoculation. Can cause conjunctivitis, episcleritis, uveitis, choroiditis, and retrobulbar lesions, among others. The granulomatous lesion may be accompanied by a serous-purulent discharge, redness, and eyelid edema.
Extracutaneous	Observed in patients with acquired immunodeficiency syndrome (AIDS), diabetes, alcoholism, granulomatous diseases, cirrhosis, renal transplantation, malignancies and under corticosteroid or immunosuppressive treatment.
Pulmonary	Occurs by inhalation of propagules or hematogenous spread. Usually associated with chronic obstructive pulmonary disease, alcoholism, chronic use of corticosteroids and, immunosuppressive diseases. Presents tuberculosis-like symptoms.
Meningeal	Infrequent presentation. Usually associated with immunosuppressive diseases.
Osteoarticular	May occur by direct trauma, invasion through a preexisting cutaneous lesion or hematogenous spread. The lesions may vary from small granulomas to large lytic lesions. One or several joints and bones can be involved, as well as tenosynovitis or bursitis. In immunocompetent patients, monoarthritis is more frequent than multiple articular involvement.
Sepsis	May occur from a cutaneous focus.
Systemic or systemic disseminated	Extremely rare. Always associated with immunosuppressive diseases.
Immunoreactive	Rare occurrence, mainly in places with a large number of cases of the disease. Can produce erythema nodosum, erythema multiforme, Sweet’s syndrome, and reactive arthritis.
